# Hyperoxia Disrupts Extracellular Signal-Regulated Kinases 1/2-Induced Angiogenesis in the Developing Lungs

**DOI:** 10.3390/ijms19051525

**Published:** 2018-05-20

**Authors:** Renuka T. Menon, Amrit Kumar Shrestha, Roberto Barrios, Binoy Shivanna

**Affiliations:** 1 Section of Neonatology, Department of Pediatrics, Baylor College of Medicine, Houston, TX 77030, USA; Renuka.Menon@bcm.edu (R.T.M.); Amrit.Shrestha@bcm.edu (A.K.S.); 2 Department of Pathology and Genomic Medicine, Houston Methodist Hospital, Houston, TX 77030, USA; rbarrios@houstonmethodist.org

**Keywords:** extracellular signal-regulated kinases 1/2, hyperoxia, bronchopulmonary dysplasia, HPAECs, angiogenesis, cell cycle

## Abstract

Hyperoxia contributes to the pathogenesis of bronchopulmonary dysplasia (BPD), a chronic lung disease of infants that is characterized by interrupted alveologenesis. Disrupted angiogenesis inhibits alveologenesis, but the mechanisms of disrupted angiogenesis in the developing lungs are poorly understood. In pre-clinical BPD models, hyperoxia increases the expression of extracellular signal-regulated kinases (ERK) 1/2; however, its effects on the lung endothelial ERK1/2 signaling are unclear. Further, whether ERK1/2 activation promotes lung angiogenesis in infants is unknown. Hence, we tested the following hypotheses: (1) hyperoxia exposure will increase lung endothelial ERK1/2 signaling in neonatal C57BL/6J (WT) mice and in fetal human pulmonary artery endothelial cells (HPAECs); (2) ERK1/2 inhibition will disrupt angiogenesis in vitro by repressing cell cycle progression. In mice, hyperoxia exposure transiently increased lung endothelial ERK1/2 activation at one week of life, before inhibiting it at two weeks of life. Interestingly, hyperoxia-mediated decrease in ERK1/2 activation in mice was associated with decreased angiogenesis and increased endothelial cell apoptosis. Hyperoxia also transiently activated ERK1/2 in HPAECs. ERK1/2 inhibition disrupted angiogenesis in vitro, and these effects were associated with altered levels of proteins that modulate cell cycle progression. Collectively, these findings support our hypotheses, emphasizing that the ERK1/2 pathway is a potential therapeutic target for BPD infants with decreased lung vascularization.

## 1. Introduction

Bronchopulmonary dysplasia (BPD) is a chronic lung disease of premature infants that is characterized by interrupted lung development [1]. The incidence of BPD has remained unchanged over the past few decades, and BPD is still the most common long-term morbidity of preterm infants [2]. Importantly, there are no specific therapies for BPD. In addition, BPD is the second most expensive childhood disease after asthma. Therefore, there is a need for improved therapies to prevent and treat BPD.

Decreased alveolarization or alveolar simplification and dysmorphic lung vascularization are histopathological hallmarks of BPD [3,4]. Lung blood vessels are crucial for healthy lungs. Abnormal lung angiogenesis is a characteristic feature of BPD [5]. Lung angiogenesis facilitates alveolarization (lung development), and disrupted angiogenesis can interrupt alveolarization in the developing lungs [6]. Therefore, understanding the mechanisms that promote the development and function of the lung blood vessels is vital to prevent and treat this human disease. Toward this end, vascular endothelial growth factor (VEGF) and nitric oxide (NO) signaling pathways have been extensively investigated and have been shown to be necessary for lung development in health and disease in neonatal animals [7,8,9,10,11]. VEGF restores the alveolar and pulmonary vascular structure and function via the endothelial nitric oxide synthase pathway in experimental BPD and pulmonary hypertension (PH) [12,13,14]. However, these results were not replicated in clinical studies [15,16]. Recent evidence suggests that inhaled NO combined with vitamin A can decrease the incidence of BPD better than NO therapy alone [17]. Thus, there is a need to identify additional druggable molecular targets that can complement the inhaled NO therapy to promote the development and function of the lung vascular system.

Lung development is orchestrated by a complex process involving signaling by growth factors [18], which mediate their effects mostly via the activation of mitogen-activated protein (MAP) and phosphatidylinositol 3-OH kinases. Among the four major families of MAP kinases, the extracellular signal-regulated kinases (ERK)1/2 were shown to primarily mediate proliferation and differentiation of many cell types, whereas c-Jun NH_2_-terminal kinases and p38 kinase mainly induce cell apoptosis [19]. In fact, ERK1/2 are activated during development in many organisms [20,21] and regulate morphogenesis in several organs including the lungs [22,23,24]. Thus, it seems logical that disruption of these signaling pathways may mechanistically contribute to a developmental lung disease such as BPD.

Supplemental oxygen is frequently used as a life-saving therapy in preterm infants with respiratory failure; however, excessive oxygen exposure or hyperoxia contributes to BPD pathogenesis. We [25] and others [26,27] have demonstrated that hyperoxia-induced lung parenchymal and vascular injury in newborn mice leads to a phenotype that is similar to that of human BPD. So, we used this model to investigate the effects of hyperoxia on the expression and activation of ERK1/2 proteins in the developing lungs. ERK1/2 activation is shown to protect alveolar epithelial cells against hyperoxic injury [28,29]. However, there are several knowledge gaps, including: (1) the effects of hyperoxia on the expression and activation of ERK1/2 in neonatal mouse and fetal human lung endothelial cells; (2) the effects of ERK1/2 signaling on lung angiogenesis in preterm infants. Therefore, using neonatal C57BL/6J wild type (WT) and fetal human lung cells, we tested the following hypotheses: (1) hyperoxia exposure will increase endothelial ERK1/2 signaling in neonatal C57BL/6J (WT) mouse lungs and fetal human lung endothelial cells; (2) inhibition of ERK1/2 signaling will disrupt angiogenesis in vitro by repressing cell cycle progression.

## 2. Results

### 2.1. Hyperoxia Exposure Transiently Activates ERK1/2 in Neonatal Mouse Lungs

**Figure 1:**
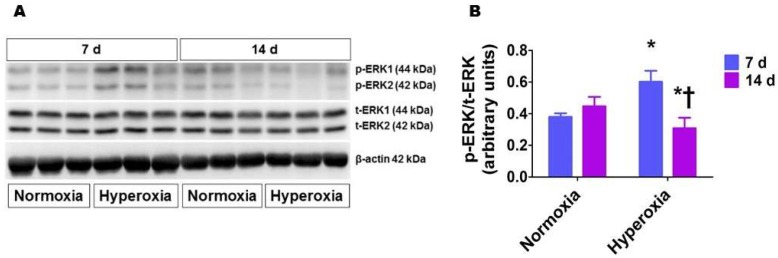
Lung phosphorylated extracellular signal-regulated kinases (ERK)1/2 protein levels in neonatal wild-type (WT) mice exposed to hyperoxia. Lung proteins obtained from neonatal WT mice exposed to 21% O_2_ (normoxia) or 70% O_2_ (hyperoxia) for up to two weeks (*n* = 6/exposure) were subjected to immunoblotting using antibodies against total ERK1/2, phosphorylated ERK1/2, or β-actin. Representative immunoblot showing total ERK1/2 and phosphorylated ERK1/2 protein expression (**A**). Densitometric analyses wherein the phosphorylated ERK1/2 band intensities were quantified and normalized to those of total ERK1/2 (**B**). The values are presented as mean ± SD. Significant differences between the normoxia and hyperoxia groups are indicated by * *p* < 0.05. Significant differences between the hyperoxia groups are indicated by † *p* < 0.001 (Two-way ANOVA).

The level of protein phosphorylation correlates strongly with the activity of a protein. Therefore, we quantified phosphorylated (p) ERK1/2 protein levels in whole lung homogenates to determine if hyperoxia activates ERK1/2. Western blot analyses (Figure 1) showed that hyperoxia exposure for one week increased ERK1/2 phosphorylation (0.6 ± 0.07 vs. 0.38 ± 0.02). However, prolonged hyperoxia exposure (14 d) decreased ERK1/2 activation compared to age-matched controls (0.31 ± 0.06 vs. 0.43 ± 0.08), suggesting that hyperoxia transiently activates ERK1/2 in the developing lungs.

### 2.2. Hyperoxia Exposure Transiently Activates ERK1/2 in Neonatal Mouse Lung Endothelial Cells

**Figure 2:**
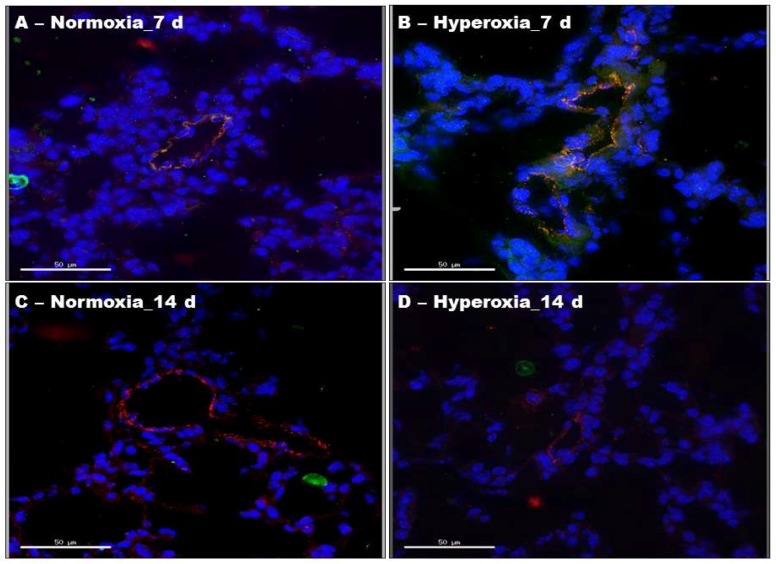
Phosphorylated ERK1/2 protein levels in lung endothelial cells of neonatal WT mice exposed to hyperoxia. One-day-old WT mice were exposed to either 21% O_2_ (normoxia) or 70% O_2_ (hyperoxia) for one or two weeks (*n* = 6/exposure/time-point), after which lung sections were processed for colocalization studies. (**A**–**D**) Representative merged images of lung sections stained with anti-pERK1/2 (green) and anti-vWF (red) antibodies, and DAPI (blue). Scale bar = 50 µM.

To investigate if hyperoxia activates ERK1/2 in mouse lung endothelial cells, we performed immunofluorescence colocalization experiments using lung sections from neonatal mice exposed to normoxia or hyperoxia for one or two weeks. We localized pERK1/2 protein expression in endothelial cells by immunofluorescence labelling using anti-pERK1/2 and anti-von Willebrand factor (vWF) antibodies. Figure 2B shows a clear overlap between the green (pERK1/2) and red (vWF) signals, indicating that pERK1/2 is expressed in lung endothelial cells. Similar to our results of immunoblotting experiments with whole lung homogenates, hyperoxia increased ERK1/2 activation in lung endothelial cells compared with the normoxia group at one week of life (Figure 2B). However, analyses of the time-dependent effects of hyperoxia revealed that ERK1/2 activation significantly decreased in the hyperoxia group at two weeks of life (Figure 2D), a time point at which lung development is still occurring.

### 2.3. Prolonged Hyperoxia Exposure Interrupts Pulmonary Vascularization in Neonatal Mice

**Figure 3:**
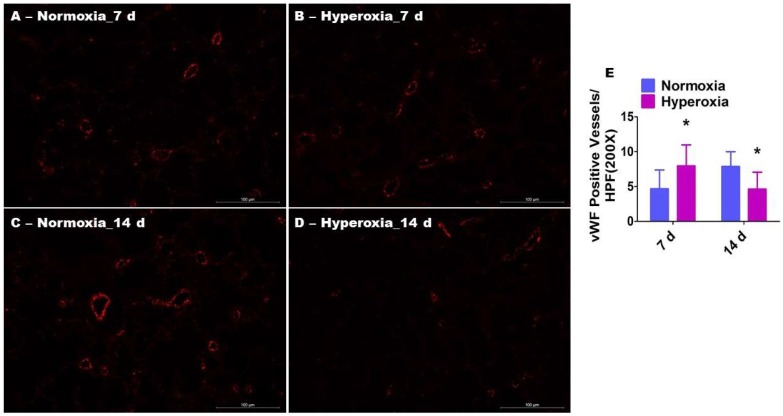
Pulmonary vascularization in neonatal WT mice exposed to hyperoxia. One-day-old WT mice were exposed to either 21% O_2_ (normoxia) or 70% O_2_ (hyperoxia) for one or two weeks (*n* = 6/exposure/time-point), following which the lung sections were stained with anti-von Willebrand factor (vWF) antibodies. (**A**–**D**) Representative vWF-stained lung blood vessels (red). (**E**) Quantitative analysis of vWF-stained lung blood vessels per high-power field (HPF). The values are presented as the mean ± SD. Two-way ANOVA analysis showed an effect of hyperoxia and duration of exposure and an interaction between them for the dependent variable, vWF-stained vessels, in this figure. Significant differences between normoxia- and hyperoxia-exposed mice are indicated by * *p* < 0.01 (Two-way ANOVA). Scale bar = 100 µM.

We next determined pulmonary vascularization by quantifying the vWF-stained lung blood vessels. Interestingly, the changes in pulmonary vascularization followed an pattern identical to that of lung endothelial cell ERK1/2 activation in hyperoxia-exposed animals. One week of hyperoxia exposure (Figure 3A,B,E) significantly increased the number of vWF-stained lung blood vessels (7.3 ± 3.4 vs. 4.6 ± 2.7), whereas prolonged (two weeks) hyperoxia exposure (Figure 3C–E) decreased the number of blood vessels (4.9 ± 2.4 vs. 7.4 ± 2.4) in comparison with normoxia-exposed mice.

### 2.4. Hyperoxia Exposure Increases Pulmonary Endothelial Cell Apoptosis in Neonatal Mice

**Figure 4:**
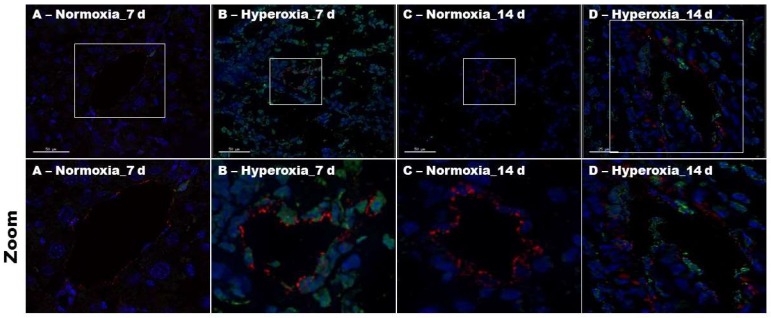
Lung endothelial cell apoptosis in neonatal WT mice exposed to hyperoxia. One-day-old WT mice were exposed to either 21% O_2_ (normoxia) or 70% O_2_ (hyperoxia) for one or two weeks (*n* = 6/exposure/time-point), after which the lung sections were processed for colocalization studies. (**A**–**D**) Representative merged images of lung sections stained with TUNEL (green), anti-vWF (red) antibody, and DAPI (blue). The frames in the original magnification figures represent the zoomed regions. Scale bar = 50 µM.

To identify the mechanisms through which hyperoxia interrupts lung angiogenesis, we performed immunofluorescence colocalization experiments using lung sections from neonatal mice exposed to normoxia or hyperoxia for one or two weeks. We determined apoptosis in lung endothelial cells by immunofluorescence labelling using an indirect TUNEL assay and anti-vWF antibodies. Figure 4 shows an increased intensity of apoptotic stain (green) in vWF-stained endothelial cells (red) in animals exposed to hyperoxia (Figure 4B,D), indicating that hyperoxia causes lung endothelial cell apoptosis.

### 2.5. Hyperoxia Exposure Activates ERK1/2 in HPAECs

**Figure 5:**
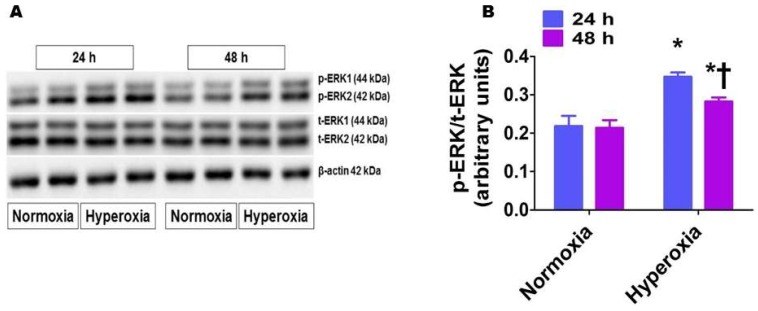
Phosphorylated ERK1/2 protein levels in human pulmonary artery endothelial cells (HPAECs) exposed to hyperoxia. HPAECs were exposed to normoxia or hyperoxia for 24 or 48 h, following which whole-cell proteins were extracted, and immunoblotting was performed using antibodies against total ERK1/2, phosphorylated ERK1/2, or β-actin. Representative immunoblot showing total ERK1/2 and phosphorylated ERK1/2 protein expression (**A**). Densitometric analyses wherein the phosphorylated ERK1/2 band intensities were quantified and normalized to those of total ERK1/2 (**B**). The values are presented as mean ± SD (*n* = 6/group). Two-way ANOVA analysis showed an effect of hyperoxia and duration of exposure and an interaction between them for the dependent variable, p-ERK1/2, in this figure. Significant differences between normoxia- and hyperoxia-exposed cells are indicated by * *p* < 0.001. Significant differences between hyperoxia-exposed cells are indicated by † *p* < 0.01 (Two-way ANOVA).

To examine the clinical significance of our findings, we used fetal HPAECs to determine if hyperoxia similarly activates ERK1/2 in the developing lungs of preterm infants. Similar to our findings in neonatal mouse lungs, time-dependent studies showed that hyperoxia increased ERK1/2 phosphorylation (0.35 ± 0.01 vs. 0.22 ± 0.03), and the extent of phosphorylation declined (0.28 ± 0.01 vs. 0.21 ± 0.02) as the duration of hyperoxia was prolonged (Figure 5).

### 2.6. PD98059 Efficiently Inhibits ERK1/2 Activation in HPAECs

**Figure 6:**
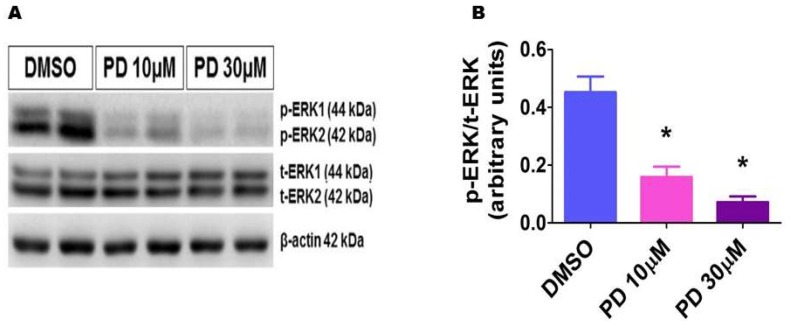
Decreased phosphorylated ERK1/2 protein levels in HPAECs treated with PD98059. HPAECs were treated with dimethylsulfoxide (DMSO) or PD98059 at concentrations of 10 (PD 10) or 30 (PD 30) µM for 30 min, after which whole-cell proteins were extracted, and immunoblotting was performed using antibodies against total ERK1/2, phosphorylated ERK1/2, or β-actin. Representative immunoblot showing total ERK1/2 and phosphorylated ERK1/2 protein expression (**A**). Densitometric analyses wherein the phosphorylated ERK1/2 band intensities were quantified and normalized to those of total ERK1/2 (**B**). The values are presented as mean ± SD (*n* = 6/group). Significant differences between DMSO- and PD-treated cells are indicated by * *p* < 0.001 (One-way ANOVA).

To investigate if ERK1/2 plays a direct role in angiogenesis in the developing lungs, we performed in vitro angiogenesis assays after inhibiting ERK1/2 activity by PD98059 in HPAECs. To this end, we first validated ERK1/2 inhibition by determining the expression of phosphorylated ERK1/2 protein levels in PD98059-treated cells. As expected, PD98059 decreased phosphorylated ERK1/2 protein expression in a dose-dependent manner (Figure 6), indicating that PD98059 efficiently inhibits ERK1/2 activation also in these cells.

### 2.7. ERK1/2 Inhibition Decreases HPAEC Migration

**Figure 7:**
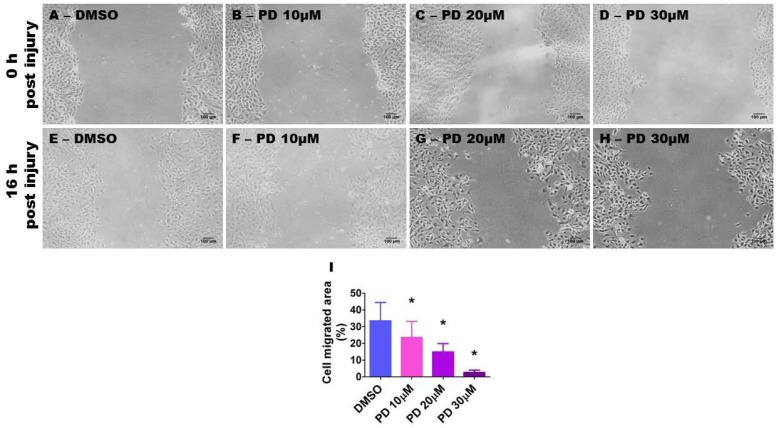
Suppression of ERK1/2 activity decreases HPAEC migration. HPAECs grown as monolayers in six-well plates were treated with 10 µg/mL of mitomycin for 2 h and scratched with a 200 µL pipette tip. The cells were then treated with dimethylsulfoxide (DMSO) or PD98059 at concentrations of 10 (PD 10), 20 (PD 20), or 30 (PD 30) µM. The wound closure area was analyzed using Image J software after 16 h of treatment. (**A**–**H**) Representative photographs showing cell migration. (**I**) Quantitative analysis of cell migration. The values are presented as mean ± SD (*n* = 6/group). Significant differences between DMSO- and PD-treated cells are indicated by *, (DMSO vs. PD 10 [*p* < 0.05]; DMSO vs. PD 20 and PD 30 [*p* < 0.001]) (One-way ANOVA). Scale bar = 100 µM.

Endothelial cell migration, proliferation, and tubule formation are essential steps in angiogenesis. To determine the effects of ERK1/2 inhibition on these steps, we performed scratch, proliferation, and tubule formation assays in cells treated with varying concentrations of PD98059. We initially performed the scratch assay and quantified cell migration by assessing the extent of wound closure in mitomycin-treated cell monolayers exposed to the vehicle or PD98059. The extent of wound closure was significantly decreased by PD98059 in a dose-dependent manner (Figure 7), indicating that ERK1/2 inhibition decreases cell migration. When compared with vehicle-treated cells, 10, 20, and 30 µM of PD98059 inhibited cell migration by 29.6, 55.5, and 91.8%, respectively (Figure 7I).

### 2.8. ERK1/2 Inhibition Decreases HPAEC Proliferation

**Figure 8:**
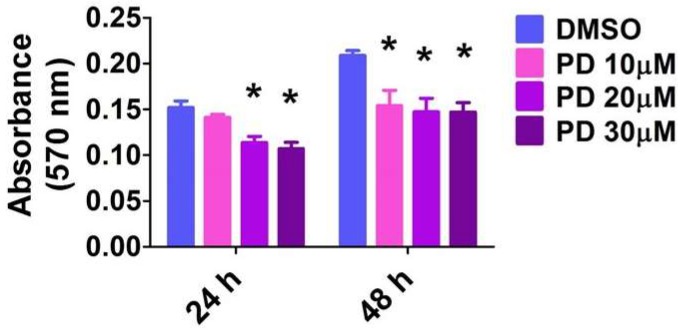
Suppression of ERK1/2 activity decreases HPAEC proliferation. HPAECs were treated with dimethylsulfoxide (DMSO) or PD98059 at concentrations of 10 (PD 10), 20 (PD 20), or 30 (PD 30) µM for 24 or 48 h, following which cell proliferation was assessed by the MTT (3-(4,5-dimethylthiazolyl-2)-2,5-diphenyltetrazolium bromide) assay. The values are presented as mean ± SD (*n* = 10/group). Two-way ANOVA analysis showed an effect of PD and duration of exposure and an interaction between them for the dependent variable, absorbance at 570 nm, in this figure. Significant differences between DMSO- and PD-treated cells are indicated by * *p* < 0.001 (Two-way ANOVA).

The MTT activity reflects the cell number, and, thus, the measured absorbance positively correlates with cell proliferation. As expected, there was a time-dependent effect on cell proliferation in vehicle-treated cells. Their proliferation rate increased by 38% in 24 h (Figure 8). However, PD98059 decreased cell proliferation in a dose-dependent manner (Figure 8). When compared with vehicle-treated cells, 10, 20, and 30 µM of PD98059 inhibited cell proliferation by 6.7, 26.4, and 29.1%, respectively, at 24 h and by 25, 29, and 30.8%, respectively, at 48 h.

### 2.9. ERK1/2 Inhibition Decreases HPAEC Tubule and Mesh Formation

**Figure 9:**
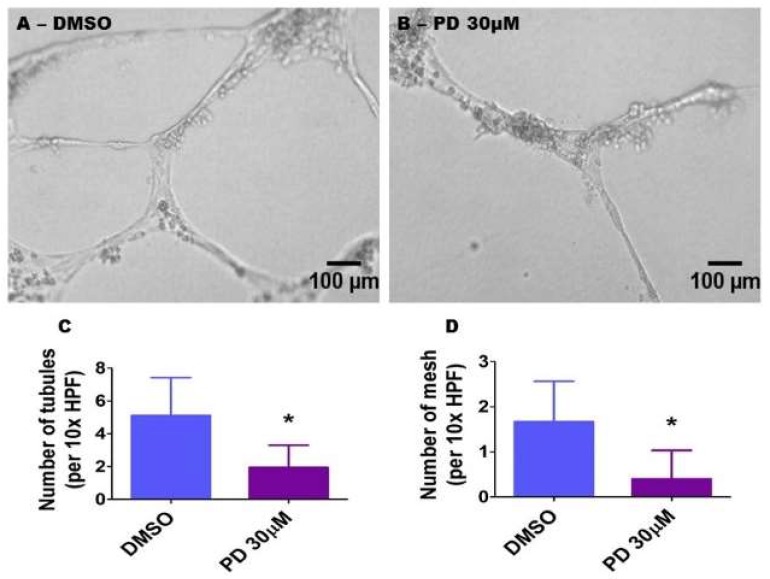
Suppression of ERK1/2 activity decreases HPAEC tubule and mesh formation. HPAECs were pre-treated with dimethylsulfoxide (DMSO) or 30 µM PD98059 (PD 30) for 30 min before being loaded on growth factor-reduced Matrigel (BD Bioscience) in 96-well plates. Following an incubation period of 18 h, tubule formation was quantified. (**A**,**B**) Representative photographs showing tubule formation in growth factor-reduced Matrigel. (**C**,**D**) Quantitative analysis of tubule (**C**) and mesh (**D**) formation. The values are presented as mean ± SD (*n* = 9/group). Significant differences between DMSO- and PD-treated cells are indicated by * *p* < 0.001 (*t*-test). Scale bar = 100 µM.

A Matrigel assay was performed to determine the extent of tubule and mesh formation in cells treated with the vehicle or with 30 µM PD98059. Consistent with its effects on cell proliferation and migration, PD98059 decreased HPAEC tubule (1.9 ± 1.3 vs. 5.1 ± 2.3) (Figure 9A–C) and mesh (0.4 ± 0.6 vs. 1.7 ± 0.9) (Figure 9A,B,D) formation, in comparison with vehicle-treated cells.

### 2.10. ERK1/2 Inhibition Alters the Level of Proteins That Regulate Cell Cycle Progression in HPAECs

**Figure 10:**
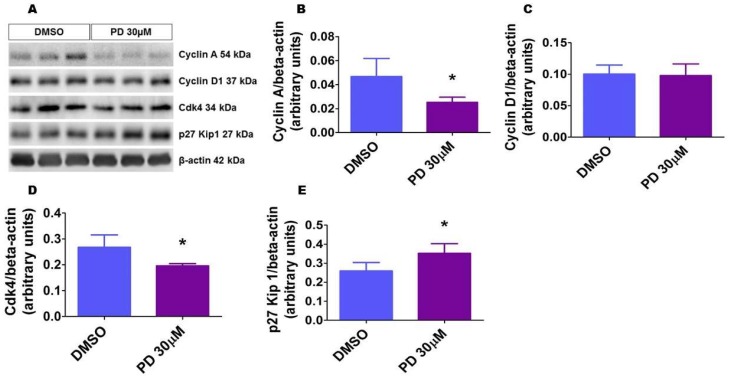
ERK1/2 inhibition affects the expression of cell cycle regulatory proteins. HPAECs were treated with dimethylsulfoxide (DMSO) or 30 µM PD98059 (PD 30) for 24 h, after which whole-cell protein were extracted, and immunoblotting was performed using antibodies against the following proteins: cyclin A, cyclin D, Cdk4, p27, and β-actin. Representative immunoblots showing the expression of the above proteins (**A**). Densitometric analyses wherein cyclin A (**B**), cyclin D (**C**), Cdk4 (**D**), and p27 Kip 1 (**E**) band intensities were quantified and normalized to those of total β-actin. The values are presented as mean ± SD (*n* = 6/group). Significant differences between DMSO- and PD-treated cells are indicated by * *p* < 0.05 (*t*-test).

We finally investigated the mechanisms by which ERK1/2 inhibition disrupts angiogenesis in vitro. Because cell cycle regulation is the major biological pathway affected by ERK1/2 signaling, we determined the effects of ERK1/2 inhibition on the expression of proteins that modulate the crucial G1/S phase of cell cycle. Cyclins A and D and Cdk4 promote G1/S phase transition, while the cyclin-dependent kinase inhibitor p27 prevents this transition. Exposure of cells to the ERK1/2 inhibitor for 24 h decreased the protein levels of cyclin A and Cdk4 and increased the protein levels of p27. Cyclin D expression was similar between the vehicle- and PD-treated cells (Figure 10). These findings indicate that repression of cell cycle progression is one of the mechanisms by which ERK1/2 inhibition interrupts angiogenesis.

## 3. Discussion

In this study, we investigated the interaction between hyperoxia and ERK1/2 activation in mouse lungs and fetal HPAECs. Previously, we demonstrated that moderate hyperoxia exposure leads to increased lung oxidative stress and inflammation and causes alveolar and pulmonary vascular simplification, pulmonary vascular remodeling, and PH [25]. Further, we showed in the same model that exposure of mice to neonatal hyperoxia causes lung developmental abnormalities that persist into adolescence [30]. These findings indicate that the phenotype of our mouse model closely aligns with that of preterm infants with BPD and PH. Therefore, we chose the same model for this study and found that hyperoxia exposure transiently increases ERK1/2 activation before decreasing it to below-baseline levels. Further, we show that hyperoxia exposure affects mouse lung vascularization in an identical pattern. Finally, using fetal HPAECs, we demonstrate that inhibition of ERK1/2 pathway disrupts angiogenesis by repressing cell cycle progression.

Lung angiogenesis actively contributes to alveologenesis during development, and healthy lung blood vessels are necessary to maintain the structural and functional integrity of alveolar structures later in life. Numerous in vitro studies have demonstrated that ERK1/2 promote angiogenesis. For example, Mavria et al. [31] demonstrated that these kinases promote endothelial cell survival and sprouting by repressing Rho kinase signaling. Similarly, Murphy et al. [32] showed that sorafenib exerts tumoricidal anti-angiogenic effects by inhibiting of ERK1/2 signaling. Further, an elegant in vivo study using mice lacking endothelial ERK1/2 genes demonstrated that ERK1/2 genes are necessary for embryonic angiogenesis [33]. Therefore, we initially determined the effects of hyperoxia on ERK1/2 activation in the lungs. Hyperoxia exposure was shown to increase ERK1/2 activation in whole lung homogenates and lung epithelial cells by several investigators [34,35,36,37,38,39]; however, the effects of hyperoxia on lung endothelial cell ERK1/2 activation are poorly understood. Our study demonstrates that hyperoxia exposure increases lung endothelial cell ERK1/2 activation at postnatal day (PND) 7, but decreases their activation at PND14. Importantly, PND14 is still a critical time period for lung development in mice. Lung development occurs at an accelerated rate between PND5 and PND14, and the maximal alveolar number is reached by PND39 [40]. These findings indicate that there may be a mechanistic link between endothelial ERK1/2 signaling and hyperoxia-induced developmental lung injury. To this end, we determined the time-dependent effects of hyperoxia on pulmonary vascularization in our mouse model.

Brief exposures to a high inspired O_2_ concentration (>95% O_2_) or prolonged exposures to a moderate oxygen concentration was shown to inhibit lung angiogenesis. Here, we show that exposure to a moderate oxygen concentration (70% O_2_) initially increased and later induced a significant decline in angiogenesis. Interestingly, hyperoxia-induced changes in lung vascularization paralleled those of ERK1/2 activation. Further, the decreased ERK1/2 activation correlated with increased apoptosis in lung endothelial cells. It is possible that upon exposure to moderate hyperoxia, several angiogenic molecules, such as ERK1/2, are activated to promote or maintain angiogenesis and facilitate lung repair. However, with prolonged hyperoxia exposure, these changes are not sustained to promote healing and prevent further damage from hyperoxic injury. Therefore, we hypothesized that the early ERK1/2 activation upon hyperoxia exposure is an adaptive response to mitigate rather than to potentiate hyperoxic lung injury. To test this hypothesis and to examine the clinical significance of our animal studies, we investigated the effects of hyperoxia exposure on ERK1/2 activation and the effects of ERK1/2 inhibition on angiogenesis using fetal HPAECs. HPAECs were selected because: (1) their proliferation and maturation are crucial for alveolarization and lung growth; (2) their dysfunction contributes to BPD pathogenesis; (3) arterial endothelial cells are enriched in ERK1/2 proteins. Similar to our findings in mouse lungs, hyperoxia exposure transiently increased ERK1/2 activation before decreasing it, when compared with normoxia exposure. Activation of ERK1/2 promotes cell proliferation and differentiation in health [41] and protects against cell death in pathological states [42,43,44]. However, it is unclear if ERK1/2 signaling attenuates or potentiates hyperoxia-mediated cytotoxicity. Several investigators have demonstrated that ERK1/2 activation protects lung epithelial cells against hyperoxia-induced cell death [28,29,45,46]. Similarly, Ahmad et al. [47] showed that ERK1/2 activation protects adult human lung pulmonary microvascular endothelial cells against hyperoxia-induced cell death. On the other hand, Zhang et al. [36] have demonstrated that inhibition of ERK1/2 signaling decreases cytochrome c release, caspase-9 and -3 activation, and poly (ADP-ribosyl) polymerase cleavage and attenuates lung epithelial cell death in hyperoxic conditions. Similarly, Carnesecchi et al. [46] demonstrated that NADPH oxidase 1 inhibition decreases oxidative stress-mediated ERK1/2 activation and attenuates acute hyperoxic lung injury in adult mice. Further, it was also shown that ERK1/2 activation potentiates hyperoxia-induced developmental lung injury, primarily by regulating the proliferation and differentiation of fibroblasts [35]. The fate of alveolar interstitial fibroblasts influences lung epithelial proliferation and differentiation, i.e., lung development. Differentiation of alveolar interstitial fibroblasts into lipofibroblast promotes lung epithelial proliferation and differentiation [48,49], whereas their differentiation into myofibroblasts interrupts lung development [49]. Under hyperoxic conditions, ERK1/2 activation was shown to be associated with increased myofibroblast differentiation [35]. It is possible that the biological response of ERK1/2 activation may be dependent upon several factors, including the cell and tissue types, magnitude and duration of ERK1/2 activation, and the interactions between ERK1/2 and other activated pathways. Further studies using ERK1/2 transgenic mice are needed to address these knowledge gaps.

The proangiogenic effects of ERK1/2 signaling is well established in the field of cancer biology. However, it is important to note that endothelial cells display substantial organ- and tissue-specific diversity [50,51]. Further, whether ERK1/2 signaling regulates angiogenesis in the developing lungs of humans needs to be determined. Consequently, we inhibited ERK1/2 activation by PD98035 and determined the resulting effects on in vitro angiogenesis using fetal HPAECs. Angiogenesis is a highly coordinated multistep process that includes cell migration, proliferation, and tubule formation [52]. Inhibition of any of these processes disrupts angiogenesis [53]. We observed that ERK1/2 inhibition decreased fetal lung endothelial cell migration, proliferation, and tubule and mesh formation, indicating that ERK1/2 signaling regulates angiogenesis in the developing lungs. Others have shown that ERK1/2 activation is associated with pulmonary vascular development [54]; however, to the best of our knowledge, ours is the first study to indicate that ERK1/2 signaling is necessary for pulmonary vascular development. The angiogenic molecule VEGF promotes lung angiogenesis via ERK1/2 activation [54], but the downstream effectors of ERK1/2 activation are poorly characterized. Hence, we finally investigated the mechanisms by which ERK1/2 signaling regulates lung angiogenesis. A large body of evidence indicate that cell cycle regulation is the predominant biological pathway regulated by ERK1/2 signaling. G1/S phase transition is critical for cell cycle progression. Cyclins A and D and Cdk4 promote this transition, whereas cyclin-dependent kinase inhibitor p27 prevents this transition [55]. Importantly, cyclin A regulates cell cycle progression at multiple levels and therefore can be considered a master regulator of the cell cycle [56]. The Cdks determine the biological activity of cyclin A. For example, cyclin A-mediated activation of Cdk2 promotes G1/S transition, whereas activation of Cdk1 promotes G2/M phase transition. We demonstrate that ERK1/2 inhibition prevented cell cycle progression by decreasing the expression of cyclin A and Cdk4 and increasing that of p27, a conclusion supported by other investigators [33,57,58].

The major limitation of this study is that the interactions between ERK1/2 signaling and lung angiogenesis were determined by in vitro studies. However, our studies in a clinically relevant model of hyperoxia-induced developmental lung injury suggest that there may be similar interactions in vivo. Our future studies will address this limitation by performing in vivo studies using ERK1/2 transgenic mice.

In summary, we demonstrate that exposure of neonatal mice to hyperoxia causes parallel changes in lung endothelial cell ERK1/2 activation and lung vascularization, wherein it initially increases ERK1/2 activation and lung vascularization and later induces a significant decline of these biological processes. Further, our in vitro studies using primary fetal human lung endothelial cells show that ERK1/2 signaling may be necessary for pulmonary vascular development. Our findings signify that targeting ERK1/2 signaling may be beneficial for BPD infants who have decreased lung vascularization.

## 4. Materials and Methods

### 4.1. In Vivo Experiments

#### 4.1.1. Animals

This study was approved and conducted in strict accordance with the federal guidelines for the humane care and use of laboratory animals by the Institutional Animal Care and Use Committee of Baylor College of Medicine (AN-5631, 12/12/2016). C57BL/6J wild-type (WT) mice were obtained from The Jackson Laboratory (Bar Harbor, ME, USA). Timed-pregnant mice raised in our animal facility were used for the experiments. The dams were fed standard mice food and water ad libitum, and all the experimental animals were maintained in 12 h day–night cycles.

#### 4.1.2. Hyperoxia Experiments

Within 24 h of birth, WT dams and their male and female pups were exposed to 21% O_2_ (normoxia, *n* = 18) or 70% O_2_ (hyperoxia, *n* = 18) for up to two weeks. The dams were rotated between normoxia- and hyperoxia-exposed litters every 24 h during the experiment to prevent oxygen toxicity in the dams and to control for the maternal effects between the groups. Oxygen exposures were conducted in plexiglass chambers, and the animals were monitored as described previously [59].

#### 4.1.3. Lung Tissue Harvest and Protein Extraction

The lungs from a subset of study animals (*n* = 6/exposure) were snap-frozen in liquid nitrogen and stored at −80 °C for the subsequent isolation of total proteins. A mortar and pestle were used to homogenize the lung tissue in a buffer containing 50 mM Tris-HCL (pH 7.5), 0.5 M KCL, 1 M MgCL, and 0.5 M EDTA. The homogenates were centrifuged at 2400× *g* for 5 min at 4 °C. The supernatants (protein lysate) were stored at −80 °C.

#### 4.1.4. Western Blot Assays

The protein lysates from the experimental animals were separated by 10% SDS-polyacrylamide gel electrophoresis and transferred to polyvinylidene difluoride membranes. The membranes were incubated overnight at 4 °C with the following primary antibodies: anti-β-actin (Santa Cruz Biotechnologies, Santa Cruz, CA, USA; sc-47778, dilution 1:1000), anti-total ERK1/2 (Cell Signaling, Danvers, MA, USA; 4695, dilution 1:1000), and anti-phospho-ERK1/2 (Cell Signaling, Danvers, MA, USA; 9106, dilution 1:1000). The primary antibodies were detected by incubation with the appropriate horseradish peroxidase-conjugated secondary antibodies. The immunoreactive bands were detected by chemiluminescence methods, and the band densities were quantified by Image lab 5.2.1 software (Bio-Rad Laboratories, Inc., Hercules, CA, USA).

#### 4.1.5. Tissue Preparation for Immunofluorescence and Lung Vascular Morphometry Studies

A separate group of pups were euthanized at one and two weeks of life (*n* = 6/exposure/time-point), and their lungs were inflated and fixed via the trachea with 10% formalin at 25 cm H_2_O pressure for at least 10 min. Sections of the paraffin-embedded lungs were obtained for immunofluorescence studies and for the analysis of lung vascularization, as described previously [59].

#### 4.1.6. Immunofluorescence Studies

We performed double immunostaining with anti-pERK1/2 and anti-von Willebrand factor (vWF; endothelial specific marker) antibodies to localize ERK1/2 activation in the lung endothelial cells. Fresh frozen lung tissues were incubated with 7.5% normal donkey serum for 1 h to block nonspecific protein binding, after which they were incubated overnight at 4 °C with the following primary antibodies: anti-phospho-ERK1/2 (Cell Signaling, Danvers, MA, USA; 4370, dilution 1:150) and anti-vWF (Abcam, Cambridge, MA, USA; ab11713, dilution 1:50). The primary anti-pERK1/2 and anti-vWF antibodies were detected by incubation with fluorescein-conjugated donkey anti-rabbit (Alexa Fluor 488, dilution 1:200) and donkey anti-sheep (Alexa Fluor 633, dilution 1:200) secondary antibodies, respectively. An indirect TUNEL assay was used to detect apoptosis using the ApopTag Fluorescein In Situ Apoptosis detection kit (MilliporeSigma, St. Louis, MO, USA; S7110), as per the manufacturer’s recommendations. The localization of the apoptotic process in the endothelial cells was determined using vWF antibodies, as described above. All the slides were counterstained with 4′,6-diamidino-2-phenylindole (DAPI) and analyzed by confocal microscopy. The observers analyzing these slides were masked to the experimental conditions.

#### 4.1.7. Analyses of Pulmonary Vascularization

Pulmonary vessel density was determined in these animals on the basis of the immunofluorescence staining with anti-vWF antibody (Abcam, Cambridge, MA, USA; ab11713, dilution 1:50), which is an endothelial-specific marker [60]. At least 10 counts from 10 random non-overlapping fields (original magnification, 200×) were performed for each animal (*n* = 6/exposure/time-point). The observers performing these measurements were masked to the slide identity.

### 4.2. In Vitro Experiments

#### 4.2.1. Cell Culture

The human pulmonary artery endothelial cells (HPAECs) derived from the lungs of human fetus (26 weeks gestational age) were obtained from ScienCell research laboratories (San Diego, CA, USA; 3100). HPAECs were grown in 95% air and 5% CO_2_ at 37 °C in specific endothelial cell medium, according to the manufacturer’s protocol. Briefly, the cells were grown in fibronectin-coated plates containing basal endothelial cell medium supplemented with fetal bovine serum, antibiotics, and an endothelial cell growth supplement in a humidifier containing 5% CO_2_ at 37 °C. When the cell culture reached >90% confluence, the cells were subcultured with a split ratio of 1:3. Cells between passages 5–7 were used for all our experiments.

#### 4.2.2. Hyperoxia Exposure

Hyperoxia experiments were conducted in a plexiglass, sealed chamber into which a mixture of 95% O_2_ and 5% CO_2_ was circulated continuously. The chamber was placed in a Forma Scientific water-jacketed incubator at 37 °C. Once the O_2_ level inside the chamber reached 95%, the cells were placed inside the chamber for up to 48 h [61]. The cells were harvested at 24 h and 48 h of exposure to determine the effects of hyperoxia on ERK1/2 activation.

#### 4.2.3. Cell Treatment

HPAECs were treated with either 0.01% *v*/*v* dimethyl sulfoxide (DMSO) (Sigma-Aldrich, St. Louis, MO, USA; 276855) or the ERK1/2 inhibitor PD98059 (Sigma-Aldrich, St. Louis, MO, USA; P215) at varying concentrations up to 30 µM. The cells were then harvested to determine the effects of PD98059 on ERK1/2 activation, cell proliferation, migration, tubule formation, and expression of cell cycle regulatory proteins.

#### 4.2.4. Western Blot Assays

The cells were grown in complete medium on six-well plates to 70–80% confluence, after which they were exposed to normoxia (95% air and 5% CO_2_) or hyperoxia (95% O_2_ and 5% CO_2_) for up to 48 h. In a separate set of experiments, the cells grown on six-well plates were treated with DMSO or 30 µM PD98059 for 24 h. Following these treatments, whole-cell protein extracts were obtained by using the radio immunoprecipitation assay lysis buffer system (Santa Cruz Biotechnologies, Santa Cruz, CA, USA; sc-24948) and subjected to western blotting with the following antibodies: anti-β-actin (Santa Cruz Biotechnologies, Santa Cruz, CA, USA; sc-47778, dilution 1:1000), anti-cyclin A (Santa Cruz Biotechnologies, Santa Cruz, CA, USA; sc-751, dilution 1:1000), anti-cyclin D1 (Santa Cruz Biotechnologies, Santa Cruz, CA, USA; sc-8396, dilution 1:250), anti-cyclin-dependent kinase (Cdk) 4 (Santa Cruz Biotechnologies, Santa Cruz, CA, USA; sc-23896, dilution 1:250), anti-p27 Kip 1 (Abcam, Cambridge, MA, USA ; ab32034, dilution 1:1000), anti-total ERK1/2 (Cell Signaling, Danvers, MA, USA; 4695, dilution 1:1000), anti-phospho-ERK1/2 (Cell Signaling, Danvers, MA, USA; 9106, dilution 1:1000). The immunoreactive bands were detected and quantified as described in the “in vivo experiments” section.

#### 4.2.5. Cell Proliferation Assay

Cell proliferation was determined by a colorimetric assay based on the ability of viable cells to reduce the tetrazolium salt MTT (3-(4,5-dimethylthiazolyl-2)-2,5-diphenyltetrazolium bromide) (American Type Culture Collection, Manassas, VA, USA; ATCC 30-1010K) to formazan. HPAECs were grown in 96-well microplates overnight at a density of 5 × 10^4^ cells per well in 100 µL of complete medium, followed by an additional period of growth for 24 h in basal medium containing 0.5% fetal bovine serum (FBS). The cells were then treated with varying concentrations of PD98059 and grown under reduced serum conditions for up to 48 h, after which cell proliferation was assessed by the MTT reduction assay, as outlined in the MTT Assay protocol (American Type Culture Collection, Manassas, VA, USA). Briefly, at the end of the experiments, 10 µL of MTT reagent was added to each well, and the cells were incubated in a humidifier containing 5% CO_2_ at 37 °C for 2 h, at the end of which precipitates were visible in all wells. Following the incubation, 100 µL of detergent was added to each well, and the cells were incubated at room temperature in the dark for additional 2 h; the absorbance was measured at 570 nm. The absorbance readings are directly proportional to the number of cells.

#### 4.2.6. Scratch Assay

A scratch assay was used to quantify cell migration [62]. HPAECs were grown in complete medium in six-well plates to 70–80% confluence, followed by a period of growth for 24 h in basal medium containing 0.5% fetal bovine serum. To mitigate the effects of cell proliferation on wound closure, the cells were pre-treated with mitomycin (10 µg/mL) (MilliporeSigma, St. Louis, MO, USA; M4287) for 2 h. The cells were then scratched with a 200 µL pipette tip before they were treated with PD98059. The wound closure or cell migration area was estimated using Image J software (National Institutes of Health, Bethesda, MD, USA) by comparing the wounded areas at 0 h and 16 h.

#### 4.2.7. Tubule and Mesh Formation Assays

A Matrigel assay was used to determine tubule and mesh formation, as described previously [63,64]. HPAECs were grown in 96-well microplates at a density of 2 × 10^4^ cells per well in 100 µL of basal medium containing 0.5% FBS. The cells were pretreated with 30 µM PD98059 for 30 min and loaded on top of growth factor-reduced Matrigel (Corning, NY, USA; 356230). Following an incubation period of 18 h, tubule and mesh formation were quantified.

### 4.3. Statistical Analyses

The results were analyzed by the GraphPad Prism 5 software (La Jolla, CA, USA). The data were expressed as mean ± SD. In vivo experiments: At least three separate experiments were performed for each measurement (*n* = total animals from the three experiments). The effects of hyperoxia exposure on ERK1/2 activation were assessed by *t*-test, whereas the effects of time-point and exposure on pulmonary vascularization were assessed using two-way ANOVA. In vitro experiments: At least three separate experiments were performed for each measurement. One-way ANOVA was used to determine the dose-dependent effects of PD98059 on cell proliferation and ERK1/2 phosphorylation, while a nonparametric test (Kruskal–Wallis test) was used to determine the dose-dependent effects of PD98059 on cell migration. The effects of PD98059 on tubule and mesh formation were determined by *t*-test. A *p* value of <0.05 was considered significant.

## Abbreviations

BPD	bronchopulmonary dysplasia
Cdk	cyclin-dependent kinase
ERK	extracellular signal-regulated kinases
DAPI	4′,6-diamidino-2-phenylindole
HPAECs	human pulmonary artery endothelial cells
MAP kinase	mitogen-activated protein kinase
MTT	3-(4,5-dimethylthiazolyl-2)-2,5-diphenyltetrazolium bromide
PD	PD98059
PH	pulmonary hypertension
NO	nitric oxide
PND	postnatal day
WT	wild type
VEGF	vascular endothelial growth factor
vWF	von Willebrand factor

## References

[1] Jobe A.H. (2015). Animal models, learning lessons to prevent and treat neonatal chronic lung disease. Front. Med..

[2] Fanaroff A.A., Stoll B.J., Wright L.L., Carlo W.A., Ehrenkranz R.A., Stark A.R., Bauer C.R., Donovan E.F., Korones S.B., Laptook A.R. (2007). Trends in neonatal morbidity and mortality for very low birthweight infants. Am. J. Obstet. Gynecol..

[3] Husain A.N., Siddiqui N.H., Stocker J.T. (1998). Pathology of arrested acinar development in postsurfactant bronchopulmonary dysplasia. Hum. Pathol..

[4] Coalson J.J. (2003). Pathology of new bronchopulmonary dysplasia. Semin. Neonatol..

[5] Bhatt A.J., Pryhuber G.S., Huyck H., Watkins R.H., Metlay L.A., Maniscalco W.M. (2001). Disrupted pulmonary vasculature and decreased vascular endothelial growth factor, Flt-1, and TIE-2 in human infants dying with bronchopulmonary dysplasia. Am. J. Respir. Crit. Care Med..

[6] Thebaud B., Abman S.H. (2007). Bronchopulmonary dysplasia: Where have all the vessels gone? Roles of angiogenic growth factors in chronic lung disease. Am. J. Respir. Crit. Care Med..

[7] Jakkula M., Le Cras T.D., Gebb S., Hirth K.P., Tuder R.M., Voelkel N.F., Abman S.H. (2000). Inhibition of angiogenesis decreases alveolarization in the developing rat lung. Am. J. Physiol. Lung Cell. Mol. Physiol..

[8] Le Cras T.D., Markham N.E., Tuder R.M., Voelkel N.F., Abman S.H. (2002). Treatment of newborn rats with a vegf receptor inhibitor causes pulmonary hypertension and abnormal lung structure. Am. J. Physiol. Lung Cell. Mol. Physiol..

[9] Thebaud B., Ladha F., Michelakis E.D., Sawicka M., Thurston G., Eaton F., Hashimoto K., Harry G., Haromy A., Korbutt G. (2005). Vascular endothelial growth factor gene therapy increases survival, promotes lung angiogenesis, and prevents alveolar damage in hyperoxia-induced lung injury: Evidence that angiogenesis participates in alveolarization. Circulation.

[10] Stenmark K.R., Abman S.H. (2005). Lung vascular development: Implications for the pathogenesis of bronchopulmonary dysplasia. Annu. Rev. Physiol..

[11] Yamamoto H., Yun E.J., Gerber H.P., Ferrara N., Whitsett J.A., Vu T.H. (2007). Epithelial-vascular cross talk mediated by VEGF-A and HGF signaling directs primary septae formation during distal lung morphogenesis. Dev. Biol..

[12] Lin Y.J., Markham N.E., Balasubramaniam V., Tang J.R., Maxey A., Kinsella J.P., Abman S.H. (2005). Inhaled nitric oxide enhances distal lung growth after exposure to hyperoxia in neonatal rats. Pediatr. Res..

[13] Tang J.R., Markham N.E., Lin Y.J., McMurtry I.F., Maxey A., Kinsella J.P., Abman S.H. (2004). Inhaled nitric oxide attenuates pulmonary hypertension and improves lung growth in infant rats after neonatal treatment with a VEGF receptor inhibitor. Am. J. Physiol. Lung Cell. Mol. Physiol..

[14] Kunig A.M., Balasubramaniam V., Markham N.E., Seedorf G., Gien J., Abman S.H. (2006). Recombinant human vegf treatment transiently increases lung edema but enhances lung structure after neonatal hyperoxia. Am. J. Physiol. Lung Cell. Mol. Physiol..

[15] Allen M.C., Donohue P., Gilmore M., Cristofalo E., Wilson R.F., Weiner J.Z., Robinson K. (2010). Inhaled nitric oxide in preterm infants. Evid. Rep./Technol. Assess..

[16] Donohue P.K., Gilmore M.M., Cristofalo E., Wilson R.F., Weiner J.Z., Lau B.D., Robinson K.A., Allen M.C. (2011). Inhaled nitric oxide in preterm infants: A systematic review. Pediatrics.

[17] Gadhia M.M., Cutter G.R., Abman S.H., Kinsella J.P. (2014). Effects of early inhaled nitric oxide therapy and vitamin a supplementation on the risk for bronchopulmonary dysplasia in premature newborns with respiratory failure. J. Pediatr..

[18] Madurga A., Mizikova I., Ruiz-Camp J., Morty R.E. (2013). Recent advances in late lung development and the pathogenesis of bronchopulmonary dysplasia. Am. J. Physiol. Lung Cell. Mol. Physiol..

[19] English J., Pearson G., Wilsbacher J., Swantek J., Karandikar M., Xu S., Cobb M.H. (1999). New insights into the control of map kinase pathways. Exp. Cell Res..

[20] Gabay L., Seger R., Shilo B.Z. (1997). Map kinase in situ activation atlas during *Drosophila embryogenesis*. Development.

[21] Kashimata M., Sayeed S., Ka A., Onetti-Muda A., Sakagami H., Faraggiana T., Gresik E.W. (2000). The ERK-1/2 signaling pathway is involved in the stimulation of branching morphogenesis of fetal mouse submandibular glands by EGF. Dev. Biol..

[22] Karihaloo A., O’Rourke D.A., Nickel C., Spokes K., Cantley L.G. (2001). Differential mapk pathways utilized for hgf- and egf-dependent renal epithelial morphogenesis. J. Biol. Chem..

[23] Niemann C., Brinkmann V., Birchmeier W. (2000). Hepatocyte growth factor and neuregulin in mammary gland cell morphogenesis. Adv. Exp. Med. Biol..

[24] Kling D.E., Lorenzo H.K., Trbovich A.M., Kinane T.B., Donahoe P.K., Schnitzer J.J. (2002). MEK-1/2 inhibition reduces branching morphogenesis and causes mesenchymal cell apoptosis in fetal rat lungs. Am. J. Physiol. Lung Cell. Mol. Physiol..

[25] Reynolds C.L., Zhang S., Shrestha A.K., Barrios R., Shivanna B. (2016). Phenotypic assessment of pulmonary hypertension using high-resolution echocardiography is feasible in neonatal mice with experimental bronchopulmonary dysplasia and pulmonary hypertension: A step toward preventing chronic obstructive pulmonary disease. Int. J. Chron. Obstr. Pulm. Dis..

[26] Aslam M., Baveja R., Liang O.D., Fernandez-Gonzalez A., Lee C., Mitsialis S.A., Kourembanas S. (2009). Bone marrow stromal cells attenuate lung injury in a murine model of neonatal chronic lung disease. Am. J. Respir. Crit. Care Med..

[27] Lee K.J., Berkelhamer S.K., Kim G.A., Taylor J.M., O’Shea K.M., Steinhorn R.H., Farrow K.N. (2014). Disrupted pulmonary artery cgmp signaling in mice with hyperoxia-induced pulmonary hypertension. Am. J. Respir. Cell Mol. Biol..

[28] Xu D., Guthrie J.R., Mabry S., Sack T.M., Truog W.E. (2006). Mitochondrial aldehyde dehydrogenase attenuates hyperoxia-induced cell death through activation of ERK/MAPK and PI3K-Akt pathways in lung epithelial cells. Am. J. Physiol. Lung Cell. Mol. Physiol..

[29] Buckley S., Driscoll B., Barsky L., Weinberg K., Anderson K., Warburton D. (1999). ERK activation protects against DNA damage and apoptosis in hyperoxic rat AEC2. Am. J. Physiol..

[30] Menon R.T., Shrestha A.K., Shivanna B. (2017). Hyperoxia exposure disrupts adrenomedullin signaling in newborn mice: Implications for lung development in premature infants. Biochem. Biophys. Res. Commun..

[31] Mavria G., Vercoulen Y., Yeo M., Paterson H., Karasarides M., Marais R., Bird D., Marshall C.J. (2006). ERK-MAPK signaling opposes Rho-kinase to promote endothelial cell survival and sprouting during angiogenesis. Cancer Cell.

[32] Murphy D.A., Makonnen S., Lassoued W., Feldman M.D., Carter C., Lee W.M. (2006). Inhibition of tumor endothelial ERK activation, angiogenesis, and tumor growth by sorafenib (bay43-9006). Am. J. Pathol..

[33] Srinivasan R., Zabuawala T., Huang H., Zhang J., Gulati P., Fernandez S., Karlo J.C., Landreth G.E., Leone G., Ostrowski M.C. (2009). Erk1 and Erk2 regulate endothelial cell proliferation and migration during mouse embryonic angiogenesis. PLoS ONE.

[34] Konsavage W.M., Zhang L., Wu Y., Shenberger J.S. (2012). Hyperoxia-induced activation of the integrated stress response in the newborn rat lung. Am. J. Physiol. Lung Cell. Mol. Physiol..

[35] Sakurai R., Villarreal P., Husain S., Liu J., Sakurai T., Tou E., Torday J.S., Rehan V.K. (2013). Curcumin protects the developing lung against long-term hyperoxic injury. Am. J. Physiol. Lung Cell. Mol. Physiol..

[36] Zhang X., Shan P., Sasidhar M., Chupp G.L., Flavell R.A., Choi A.M., Lee P.J. (2003). Reactive oxygen species and extracellular signal-regulated kinase 1/2 mitogen-activated protein kinase mediate hyperoxia-induced cell death in lung epithelium. Am. J. Respir. Cell Mol. Biol..

[37] Buckley S., Barsky L., Weinberg K., Warburton D. (2005). In vivo inosine protects alveolar epithelial type 2 cells against hyperoxia-induced DNA damage through map kinase signaling. Am. J. Physiol. Lung Cell. Mol. Physiol..

[38] Papaiahgari S., Zhang Q., Kleeberger S.R., Cho H.Y., Reddy S.P. (2006). Hyperoxia stimulates an Nrf2-ARE transcriptional response via ROS-EGFR-PI3K-Akt/ERK map kinase signaling in pulmonary epithelial cells. Antioxid. Redox Signal..

[39] Mao Q., Gundavarapu S., Patel C., Tsai A., Luks F.I., De Paepe M.E. (2008). The fas system confers protection against alveolar disruption in hyperoxia-exposed newborn mice. Am. J. Respir. Cell Mol. Biol..

[40] Pozarska A., Rodriguez-Castillo J.A., Surate Solaligue D.E., Ntokou A., Rath P., Mizikova I., Madurga A., Mayer K., Vadasz I., Herold S. (2017). Stereological monitoring of mouse lung alveolarization from the early postnatal period to adulthood. Am. J. Physiol. Lung Cell. Mol. Physiol..

[41] Marshall C.J. (1995). Specificity of receptor tyrosine kinase signaling: Transient versus sustained extracellular signal-regulated kinase activation. Cell.

[42] Nguyen D.T., Kebache S., Fazel A., Wong H.N., Jenna S., Emadali A., Lee E.H., Bergeron J.J., Kaufman R.J., Larose L. (2004). Nck-dependent activation of extracellular signal-regulated kinase-1 and regulation of cell survival during endoplasmic reticulum stress. Mol. Biol. Cell.

[43] Wada T., Penninger J.M. (2004). Mitogen-activated protein kinases in apoptosis regulation. Oncogene.

[44] Hung C.C., Ichimura T., Stevens J.L., Bonventre J.V. (2003). Protection of renal epithelial cells against oxidative injury by endoplasmic reticulum stress preconditioning is mediated by ERK1/2 activation. J. Biol. Chem..

[45] Truong S.V., Monick M.M., Yarovinsky T.O., Powers L.S., Nyunoya T., Hunninghake G.W. (2004). Extracellular signal-regulated kinase activation delays hyperoxia-induced epithelial cell death in conditions of akt downregulation. Am. J. Respir. Cell Mol. Biol..

[46] Carnesecchi S., Deffert C., Pagano A., Garrido-Urbani S., Metrailler-Ruchonnet I., Schappi M., Donati Y., Matthay M.A., Krause K.H., Barazzone Argiroffo C. (2009). Nadph oxidase-1 plays a crucial role in hyperoxia-induced acute lung injury in mice. Am. J. Respir. Crit. Care Med..

[47] Ahmad S., Ahmad A., Ghosh M., Leslie C.C., White C.W. (2004). Extracellular ATP-mediated signaling for survival in hyperoxia-induced oxidative stress. J. Biol. Chem..

[48] McGowan S.E., Torday J.S. (1997). The pulmonary lipofibroblast (lipid interstitial cell) and its contributions to alveolar development. Annu. Rev. Physiol..

[49] Torday J.S., Torres E., Rehan V.K. (2003). The role of fibroblast transdifferentiation in lung epithelial cell proliferation, differentiation, and repair in vitro. Pediatr. Pathol. Mol. Med..

[50] Plendl J., Neumuller C., Vollmar A., Auerbach R., Sinowatz F. (1996). Isolation and characterization of endothelial cells from different organs of fetal pigs. Anat. Embryol..

[51] Gumkowski F., Kaminska G., Kaminski M., Morrissey L.W., Auerbach R. (1987). Heterogeneity of mouse vascular endothelium. In vitro studies of lymphatic, large blood vessel and microvascular endothelial cells. Blood Vessels.

[52] Goodwin A.M. (2007). In vitro assays of angiogenesis for assessment of angiogenic and anti-angiogenic agents. Microvasc. Res..

[53] Carmeliet P. (2000). Mechanisms of angiogenesis and arteriogenesis. Nat. Med..

[54] Pang J., Hoefen R., Pryhuber G.S., Wang J., Yin G., White R.J., Xu X., O'Dell M.R., Mohan A., Michaloski H. (2009). G-protein-coupled receptor kinase interacting protein-1 is required for pulmonary vascular development. Circulation.

[55] Sherr C.J., Roberts J.M. (1999). CDK inhibitors: Positive and negative regulators of G1-phase progression. Genes Dev..

[56] Yam C.H., Fung T.K., Poon R.Y. (2002). Cyclin A in cell cycle control and cancer. Cell. Mol. Life Sci. CMLS.

[57] Shen H., Zhou E., Wei X., Fu Z., Niu C., Li Y., Pan B., Mathew A.V., Wang X., Pennathur S. (2015). High density lipoprotein promotes proliferation of adipose-derived stem cells via S1P1 receptor and Akt, ERK1/2 signal pathways. Stem Cell Res. Ther..

[58] Ling L., Wei T., He L., Wang Y., Wang Y., Feng X., Zhang W., Xiong Z. (2017). Low-intensity pulsed ultrasound activates ERK1/2 and PI3K-Akt signalling pathways and promotes the proliferation of human amnion-derived mesenchymal stem cells. Cell Prolif..

[59] Shivanna B., Zhang W., Jiang W., Welty S.E., Couroucli X.I., Wang L., Moorthy B. (2013). Functional deficiency of aryl hydrocarbon receptor augments oxygen toxicity-induced alveolar simplification in newborn mice. Toxicol. Appl. Pharmacol..

[60] Shivanna B., Zhang S., Patel A., Jiang W., Wang L., Welty S.E., Moorthy B. (2015). Omeprazole attenuates pulmonary aryl hydrocarbon receptor activation and potentiates hyperoxia-induced developmental lung injury in newborn mice. Toxicol. Sci..

[61] Shivanna B., Chu C., Welty S.E., Jiang W., Wang L., Couroucli X.I., Moorthy B. (2011). Omeprazole attenuates hyperoxic injury in H441 cells via the aryl hydrocarbon receptor. Free Radic. Biol. Med..

[62] Liang C.C., Park A.Y., Guan J.L. (2007). In vitro scratch assay: A convenient and inexpensive method for analysis of cell migration in vitro. Nat. Protoc..

[63] Arnaoutova I., Kleinman H.K. (2010). In vitro angiogenesis: Endothelial cell tube formation on gelled basement membrane extract. Nat. Protoc..

[64] Ribatti D., Guidolin D., Conconi M.T., Nico B., Baiguera S., Parnigotto P.P., Vacca A., Nussdorfer G.G. (2003). Vinblastine inhibits the angiogenic response induced by adrenomedullin in vitro and in vivo. Oncogene.

